# Bone mineral density is negatively correlated with ulcerative colitis: a systematic review and meta-analysis

**DOI:** 10.1186/s40169-020-00270-0

**Published:** 2020-02-18

**Authors:** Tianyu Zhou, Jiaqi Pan, Bin Lai, Li Cen, Wenxi Jiang, Chaohui Yu, Zhe Shen

**Affiliations:** 1 Department of Gastroenterology, The First Affiliated Hospital, College of Medicine, Zhejiang University, Hangzhou, China; 2 People’s Hospital of Jianggan District, Hangzhou, China

**Keywords:** Bone mineral density, Inflammatory bowel disease, Ulcerative colitis, Meta-analysis

## Abstract

**Background:**

Newer epidemiological studies suggest that the incidence of ulcerative colitis might be increasing rapidly. Furthermore, osteoporosis in ulcerative colitis patients has gained great attention, but the epidemiologic evidence remains controversial. Therefore, a meta‐analysis was performed to explore the association between bone density and ulcerative colitis.

**Methods:**

Two investigators used PubMed, EMBASE and the Cochrane Library databases to identify all studies published before August 2019. Depending on the outcomes, investigators divided these studies into four groups (OR, SMD [BMD], SMD [z-score] and SMD [t-score]). To address the use of steroids, which is a major confounding factor in this analysis, another subgroup analysis of studies of steroid-free patients was conducted. Additionally, heterogeneity, sensitivity and stratified analyses were also performed.

**Results:**

A total of 13 cross-sectional studies that involved 1154 participants were included in the present meta-analysis, and three of them were included in the steroid-free subgroup analysis. The pooled OR was 6.41 (95% CI 2.59–15.87) and the pooled SMD (BMD), SMD (t-score) and SMD (z-score) were − 0.24 (95% CI − 0.44 to − 0.04), − 0.55 (95% CI − 0.72 to − 0.37), and − 0.38 (95% CI − 0.56 and − 0.19), respectively. Since steroids are a significant confounder, the pooled SMD of the steroid-free subgroup was − 0.55 (− 0.85 to − 0.25), which revealed a strong negative relationship between bone density and ulcerative colitis in steroid-free patients. Additionally, other subgroup analyses also revealed a strong relationship.

**Conclusions:**

This meta-analysis provides evidence for the potential association between ulcerative colitis and decreased bone density. It is essential for clinicians to consider bone mineral density in ulcerative colitis patients regardless of steroid-therapy.

## Background

Inflammatory bowel diseases, Crohn’s disease and ulcerative colitis are chronic idiopathic disorders that cause inflammation of the gastrointestinal tract. More than a decade ago, inflammatory bowel disease was rare in Asia. However, newer epidemiological studies have suggested that its incidence might be rapidly increasing in South America, Eastern Europe, Asia and Africa. In the past few years, inflammatory bowel disease has become a public health challenge worldwide and is associated with morbidity, mortality and substantial costs to society [1, 2].

Osteoporosis is a skeletal disease characterized by low bone density and microarchitectural deterioration of bone tissue, with a consequent increase in bone fragility and susceptibility to fracture [3]. Due to the systemic nature of osteoporosis, the associated increase in fracture risk affects virtually all skeletal sites, such as the hips and vertebra [4]. Osteoporosis remains a large burden worldwide. The challenges in the future include wider implementation of integrated systems of care, such as fracture liaison services, improvement of treatment adherence; and the establishment of effective and safe long–term treatment regimens in order to provide sustained reductions in fracture risk [5].

Recently, the association between inflammatory bowel diseases and bone mineral density (BMD) has gained great interest. However, the conclusions of these investigations have been contradictory, especially regarding the relationship between ulcerative colitis and BMD. Some studies have revealed that decreased BMD in individuals with inflammatory bowel disease is related to corticosteroid use but not the disease itself, and some studies concluded that BMD is reduced in patients with Crohn’s disease but not in patients with ulcerative colitis [6–9]. Therefore, we performed a meta-analysis to review the data obtained from related studies to investigate the potential association between ulcerative colitis and BMD, especially in steroid-free patients.

## Materials and methods

This systematic review and meta-analysis was performed following the meta-analysis of observational studies in epidemiology (MOOSE) statement guidelines [10].

### (1) Search strategy

Electronic databases, including PubMed, EMBASE and the Cochrane Library, were searched for relevant studies, and this search was independently conducted by two authors. All studies on BMD in ulcerative colitis patients were searched from database inception to August 2019. Two researchers separately searched for articles using the following terms: ((bone densities) OR (density, bone) OR (bone mineral density) OR (bone mineral densities) OR (density, bone mineral) OR (bone mineral content) OR (bone mineral contents) OR (osseous density) OR (bone density)) AND ((colitis, ulcerative) OR (idiopathic proctocolitis) OR (ulcerative colitis) OR (colitis gravis) OR (inflammatory bowel disease, ulcerative colitis type) OR (chronic ulcerative colitis) OR (colitis ulcerativa) OR (colitis ulcerosa) OR (colitis ulcerosa chronica) OR (colitis, mucosal) OR (colitis, ulcerative) OR (colitis, ulcerous) OR (colon, chronic ulceration) OR (histiocytic ulcerative colitis) OR (mucosal colitis) OR (ulcerative colorectitis) OR (ulcerative procto colitis) OR (ulcerative proctocolitis) OR (ulcerous colitis)). The references of the reviewed articles were hand-searched for additional potentially applicable studies.

### (2) Study selection

Studies were included when they met the following inclusion criteria: (1) original cross-sectional studies and case–control or cohort studies about BMD and ulcerative colitis; (2) studies that provided sufficient information to calculate the odds ratios (ORs) and 95% confidence intervals (CIs) or standardized mean differences (SMDs) and 95% CIs; (3) studies that diagnosed ulcerative colitis based on clinical, endoscopic, radiological, or histological data; (4) studies that measured BMD by dual-energy X-ray absorptiometry (DEXA), ultrasound bone density measurements, or other effective methods; and (5) studies published in English before August 2019. And studies exclusion criteria were as follows: (1) cell or animal studies, reviews, comments and letters; (2) duplicated studies; (3) research on irrelevant topics; (4) without necessary data or information. If the same samples were used in more than one study, the most complete and informative study was included.

### (3) Data extraction and quality assessment

Two investigators extracted the data from each study independently. The extracted information included the study type, first author’s name, publication year, geographical location, disease duration, study population and demographic data (age and sex), BMD measurement (site, outcome and method), and diagnosis of ulcerative colitis.

The quality of these case–control studies was assessed using the Newcastle–Ottawa scale (NOS) by two authors separately: studies with ≥ 6 stars were defined as high-quality studies. In addition, cross-sectional studies were assessed using the Agency for Healthcare Research and Quality (AHRQ). These studies were categorized as follows: high quality, 8–11; moderate quality, 4–7; and low quality, 0–3. Discrepancies were resolved by consensus.

### (4) Statistical analysis

The analyses were conducted using Stata Statistical Software (version 12.0; College Station; Texas 77845, USA) by two authors independently. According to the different types of data, the ORs or SMDs and their 95% CIs were calculated. If the outcome of the study was the number of low bone density patients, the ORs and 95% CIs were summarized, while if the outcome of the study was BMD, z-score, or t-score, the SMDs and 95% CIs were calculated. Depending on the outcomes, all the studies were divided into four groups: (OR, SMD [BMD], SMD [z-score] and SMD [t-score]). In light of the possible between-study variance due to the different study designs, methodologies and populations, random-effects models were used for high-heterogeneity groups, while fixed-effects models were used for low-heterogeneity groups.

Corticosteroid therapy can contribute to low BMD [26]. Glucocorticoids are conventional treatments for inflammatory bowel disease and are a potential factor contributing to osteoporosis in ulcerative colitis patients. Some studies have concluded that decreased BMD in inflammatory bowel disease patients is related to corticosteroid use but not the disease itself. To take this confounding factor into consideration, a subgroup analysis of studies of steroid-free patients was conducted.

Other subgroup analyses were also performed for studies, especially for high-heterogeneity groups, to identify the possible sources of heterogeneity. The statistical heterogeneity between studies was assessed using the Chi-square statistic, which was quantified by I^2^. This figure represents the percentage of the total variation accounted for by the between-study variation. For the I^2^-value, 0–25% represents insignificant heterogeneity, > 25% but ≤ 50% represents low heterogeneity, > 50% but ≤ 75% represents moderate heterogeneity, and > 75% represents high heterogeneity [11]. Furthermore, a sensitivity analysis was carried out to investigate the influence of individual studies and the stability of the results by omitting one study at a time. Publication bias was assessed using Begg’s regression asymmetry test. P < 0.05 was considered representative of statistically significant publication bias [12, 13].

## Results

### (1) Study selection and study characteristics

**Fig. 1:**
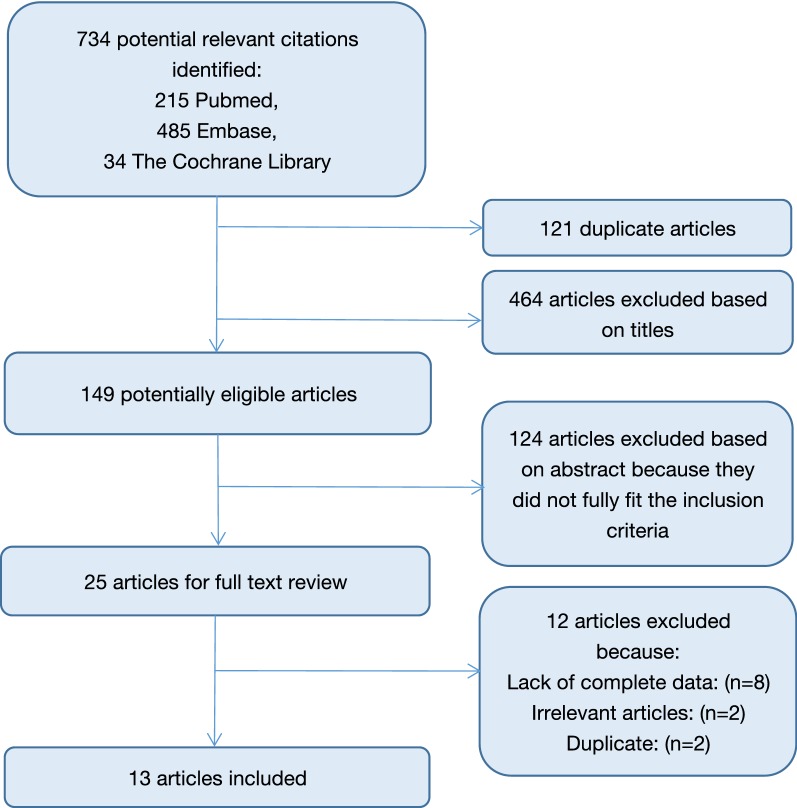
Flow diagram showing the selection of articles included in this meta-analysis

The search strategy for the meta-analysis on BMD and ulcerative colitis yielded 734 publications from PubMed, EMBASE and the Cochrane Library. Among these records, 121 publications were excluded due to duplication, and 588 articles were excluded after the screening of the titles and abstracts. Then, the full text versions of 25 articles were reviewed, and 13 articles were finally included in the present meta-analysis (Fig. 1); all of the included studies were cross-sectional studies [8, 14–25].

The main characteristics and quality assessment of all studies are listed in Table 1. The 13 selected studies included a total of 1154 participants. Among these participants, there were 570 participants in the case group and 584 participants in the control group. Among the 13 included articles, nine articles were from Europe, one article was from China, and three articles were from Brazil. In addition, 11 studies measured bone density by DEXA, while another study measured bone density by ultrasound. Of all 13 of these studies, 11 detected BMD at the lumbar spine, and six studies detected BMD at the femoral neck. Moreover, five studies calculated the number participants with low bone density, eight studies calculated the BMD (g/cm^2^), five studies calculated the t-score, and four studies calculated the z-score. Regarding the quality of these 13 cross-sectional studies, four studies were of high quality, while eight studies were of moderate quality based on the AHRQ evaluation checklist (See Appendix).

**Table 1:** Main characteristics of the included studies in this meta-analysis

Author	Country	Year	Sex M/F	Age mean age ± SD (range age) years	Disease duration mean duration ± SD	BMD measurement	Ulcerative colitis diagnosis	Detection site	Outcome	Cases	Controls	Total	Quality scores
Krela	Poland	2018	49/56	39.6 ± 15.0	7.48 ± 7.0 years	DXA	Endoscopic, histopathologic and radiologic criteria	Lumber spine, femoral neck	BMD (g/cm^2^), T score, Z score, number of low bone density	105	41	146	7
Lima	Brazil	2017	26/42	38.2 ± 9.0	None	DXA	Clinical, endoscopic, histopathologic and radiologic data	Lumber spine and femoral neck	Number of low bone density	68	67	135	7
Bastos	Brazil	2012	None	41.7 ± 14.3	None	DXA	None	Lumber spine, hip	BMD (g/cm^2^), number of low bone density	14	40	54	4
Zanetti	Brazil	2011	None	(20–50)	None	DXA	None	Lumbar spine, proximal femoral neck and total hip	Number of low bone density	20	44	64	4
Kaya	Turkey	2011	27/13	41.53 ± 11.93	38.6 ± 36.1 months	DXA	Clinical, endoscopic and histopathological data	Lumber spine, femoral neck	Number of low bone density	40	29	69	6
Pluskiewicz	Poland	2009	20/27	47.64 ± 14.83	8.6 ± 7.2 years	DXA	None	Lumber spine	BMD (g/cm^2^), T score, Z score	47	47	94	7
Liu	China	2009	None	None	50 ± 44 months	None	None	None	T score	43	37	80	3
Sakellariou	Greece	2006	Male	25.8 ± 4.6	None	Ultrasound	Histological finding	Right calcaneous	T score	14	28	42	6
Lamb	UK	2002	15/8	45	< 3 months	DXA	None	Lumber spine, femoral neck	BMD (g/cm^2^), T score, Z score	23	18	41	9
Ulivieri	Italy	2001	21/22	Male: 36.5 ± 8.4, Female: 35.3 ± 6.2	8 years	DXA	Radiologic, endoscopic and histopathological data	Lumber spine	BMD (g/cm^2^)	43	111	154	7
Schoon	The Netherlands	2000	24/20	38.4 ± 14.4	3.4 ± 7.7 months	DXA	Radiologic, endoscopic and histopathological data	Lumber spine, femoral neck	BMD (g/cm^2^)	44	44	88	9
Dinca	Italy	1999	33/16	38	8 ± 1 years	DXA	Radiologic, endoscopic and histopathological data	Lumber spine	BMD (g/cm^2^), T score	49	18	67	8
Jahnsen	Norway	1999	24/36	38	7 years	DXA	Radiologic, endoscopic and histopathological data	Lumber spine, femoral neck	BMD (g/cm^2^)	60	60	120	8

*BMD* bone mineral density, *DXA* dual energy X-ray absorptiometry

### (2) Association between BMD and ulcerative colitis

**Fig. 2:**
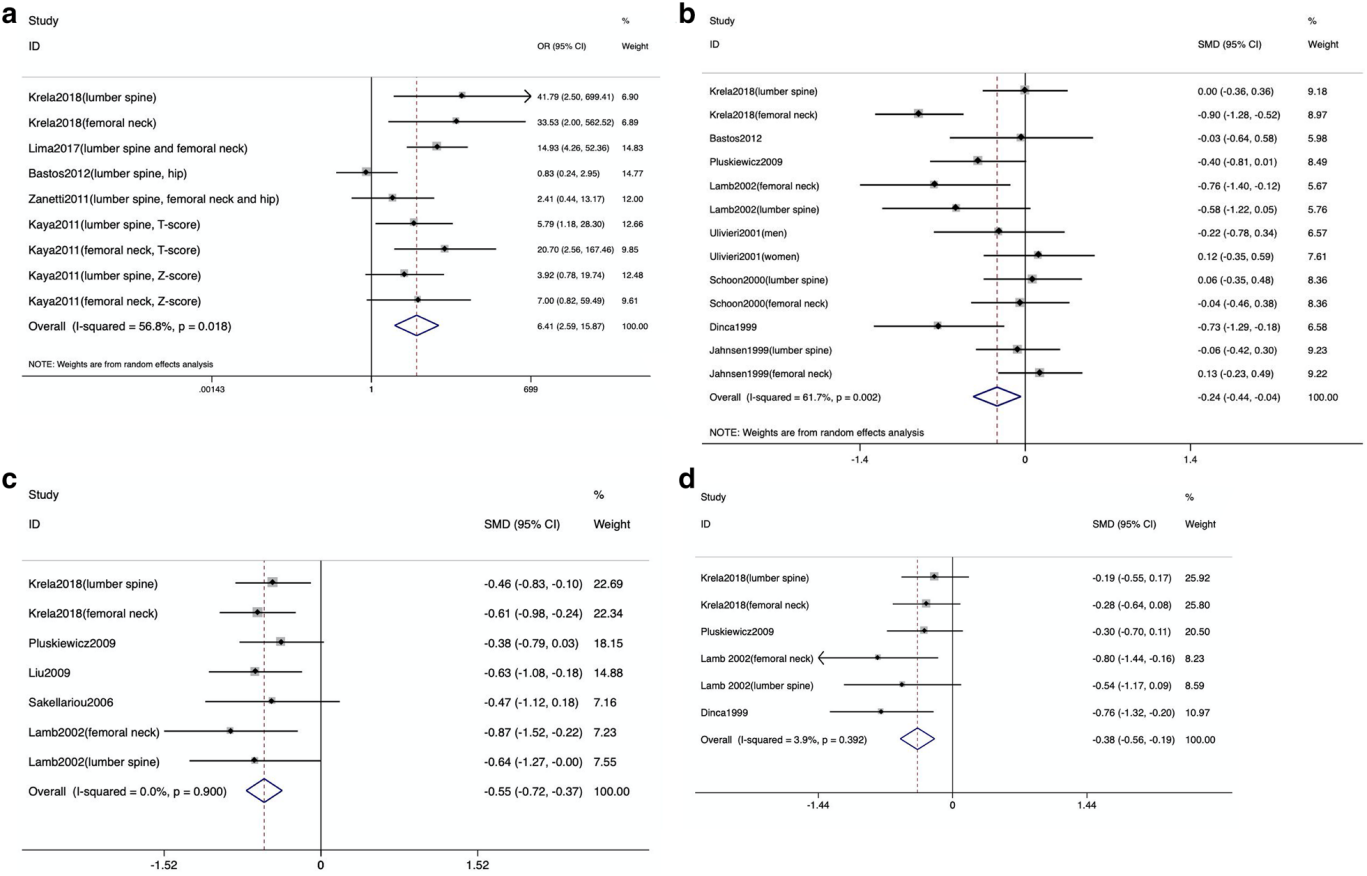
Meta-analyses of bone mineral density in ulcerative colitis patients. **A** Pooled odds ratio (OR) for the association between low bone mineral density and ulcerative colitis. The pooled OR was calculated using the random-effects model. **B** Pooled standardized mean difference (SMD) for bone mineral density in the participants. The pooled SMD was calculated using the random-effects model. **C** The SMD for the t-score in participants. The pooled SMD was calculated using the fixed-effects model. **D** The pooled SMD for the z-score in participants. The pooled SMD was calculated using the fixed-effects model

Among the four groups, ulcerative colitis patients had significantly lower BMD than healthy controls. Among the four groups, the pooled OR of low BMD was 6.41 (95% CI 2.59 to 15.87; I^2^ = 56.8%), and the pooled SMD (BMD), SMD (t-score) and SMD (z-score) were − 0.24 (95% CI − 0.44 to − 0.04; I^2^ = 61.7%), − 0.55 (95% CI − 0.72 to − 0.37; I^2^ = 0.0%), and − 0.38 (95% CI − 0.56 to − 0.19; I^2^ = 3.9%), respectively. All of these data are presented in Figs. 2. The statistical heterogeneity of the SMD (t-score) group and the SMD z-score) group had low I^2^ values of 0% and 3.9%, respectively, showing no statistically significant heterogeneity. However, the OR group and the SMD (BMD) group had moderate heterogeneity, with I^2^ values of 56.8% and 61.7%, respectively.

### (3) BMD in steroid-free ulcerative colitis patients

**Fig. 3:**
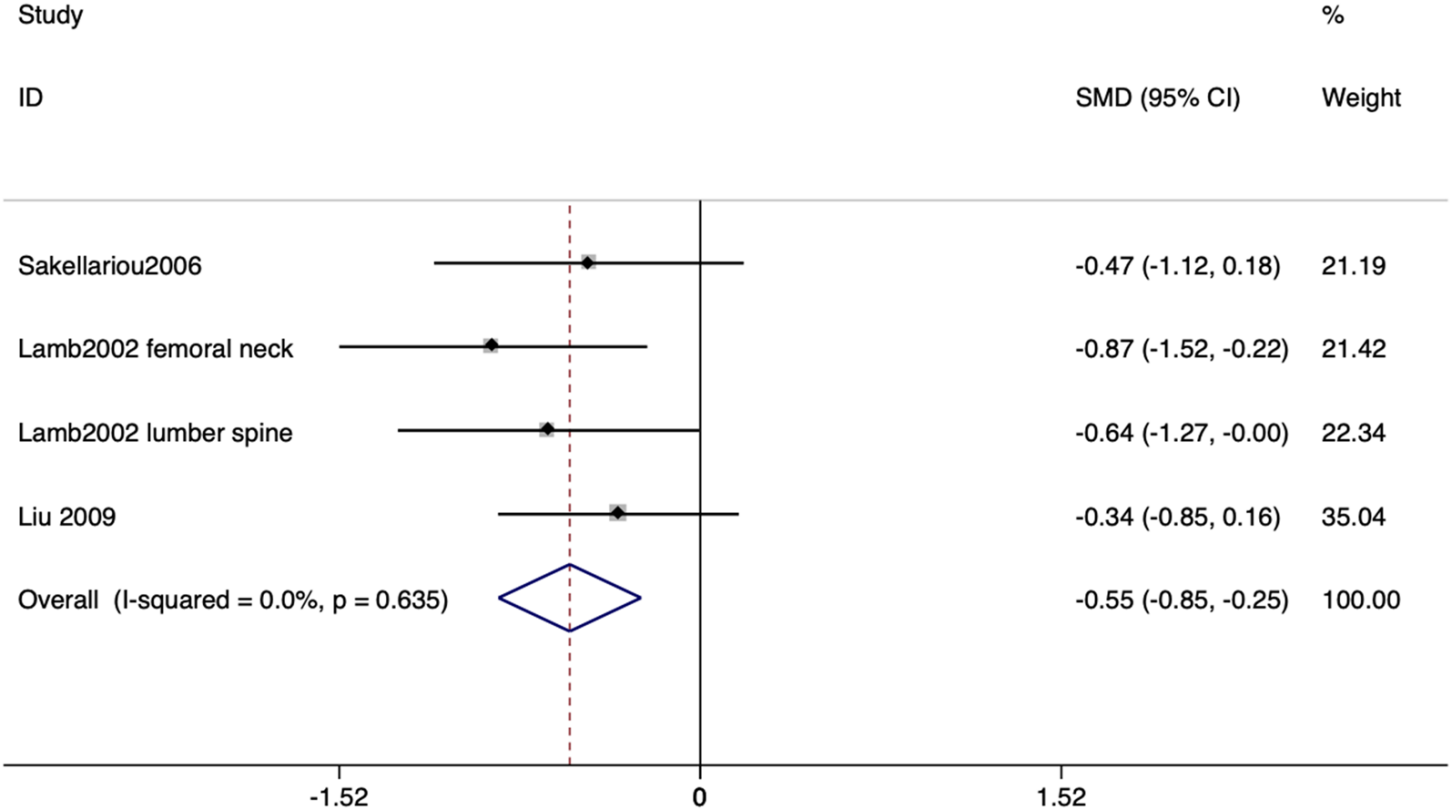
Pooled standardized mean difference (SMD) for bone mineral density and ulcerative colitis among steroid-free patients. The pooled SMD was calculated using the fixed-effects model

Among the 13 studies included in this meta-analysis, four studies [18, 20–22] analyzed BMD in steroid-free patients. In these four studies, patients have never been introduced to steroid therapy before and all of them showed a negative relationship between BMD and ulcerative colitis. The SMD depending on the t-score and its CI were calculated from three studies [20–22] because of their different outcomes. The SMD and its 95% CI was − 0.55 (− 0.85 to − 0.25; I^2^ = 0.0%), which indicated a correlation between ulcerative colitis and decreased BMD. The result is shown in Fig. 3.

### (4) Subgroup analyses

To identify the sources of heterogeneity, subgroup analyses were conducted based on detection sites, regions, ages and body mass index (BMI). The results are shown in Tables 2 and 3. Since the OR group and the SMD (BMD) group had moderate heterogeneity, subgroup analyses were conducted. When the OR group was divided into two subgroups based on the detection sites, both exhibited low heterogeneity. The ORs (95% CIs) for lumbar spine and femoral neck studies were 6.84 (95% CI 2.03 to 23.08; I^2^ = 21.3%) and 15.22 (95% CI 4.06 to 57.04; I^2^ = 0), respectively. The ORs (95% CIs) for BMI < 25 and BMI ≥ 25 studies were 37.44 (95% CI 5.10 to 274.74; I^2^ = 0) and 4.13 (95% CI 1.35 to 12.65; I^2^ = 54.3%), respectively. The SMD (BMD) group was also divided into two subgroups based on detection sites. The SMDs (95% CIs) for the lumbar spine and femoral neck were − 0.17 (95% CI − 0.35 to 0.02; I^2^ = 26.3%) and − 0.38 (95% CI − 0.91 to 0.16; I^2^ = 84%), respectively. The two subgroups based on detection sites in the OR group both exhibited low heterogeneity, which may explain the possible bias in the OR group. In all the other subgroups, the correlation between BMD and ulcerative colitis was significant, but the I^2^ of each was > 50%, which represented significant heterogeneity.

**Table 2:** Subgroup analysis of group(OR)

	No. of studies	OR (95% CI)	P	P_heterogeneity_	I^2^ (%)
Group (OR)					
Total	9	6.41 (2.59, 15.87)	< 0.001	0.018	56.8
Place					
Lumber spine	3	6.84 (2.03, 23.08)	0.002	0.281	21.3
Femoral neck	3	15.22 (4.06, 57.04)	< 0.001	0.624	0
Region					
Europe	2	37.44 (5.10, 274.74)	< 0.001	0.914	0
America	3	3.14 (0.50, 19.80)	0.223	0.005	81
Asia	4	6.72 (2.73, 16.58)	< 0.001	0.657	0
BMI (kg/m^2^)					
< 25	2	37.44 (5.10, 274.74)	< 0.001	0.914	0
≥ 25	5	4.13 (1.35, 12.65)	0.013	0.068	54.3

**Table 3:** Subgroup analyses of group [SMD (BMD)], group [SMD (t-score)] and group [SMD (z-score)]

	No. of studies	SMD (95% CI)	P	P_heterogeneity_	I^2^ (%)
Group (SMD [BMD])					
Total	13	− 0.24 (− 0.44, − 0.04)	0.021	0.002	61.7
Place					
Lumber spine	9	− 0.17 (− 0.35,0.02)	0.072	0.021	26.3
Femoral neck	4	− 0.38 (− 0.91, 0.16)	0.169	< 0.001	84
Region					
Europe	12	− 0.25 (− 0.47, − 0.04)	0.021	0.001	64.5
America	1	− 0.03 (− 0.64, 0.58)	0.918		
Average age (years old)					
< 45	10	− 0.16 (− 0.39, 0.07)	0.173	0.002	64.7
≥ 45	3	− 0.52 (− 0.83, − 0.22)	0.001	0.627	0
BMI (kg/m^2^)					
< 25	7	− 0.24 (− 0.56, 0.08)	0.139	0.001	72.4
≥ 25	4	− 0.08 (− 0.31, 0.14)	0.47	0.297	18.6
Group (SMD [T-score])					
Total	7	− 0.55 (− 0.72, − 0.37)	< 0.001	0.9	0
Place					
Lumber spine	4	− 0.50 (− 0.72, − 0.28)	< 0.001	0.827	0
Femoral neck	2	− 0.67 (− 0.99, − 0.35)	< 0.001	0.494	0
Region					
Europe	6	− 0.53 (− 0.72, − 0.34)	< 0.001	0.842	0
Asia	1	− 0.63 (− 1.08, − 0.18)	0.006		
Average age (years old)					
< 45	4	− 0.55 (− 0.76, − 0.34)	< 0.001	0.915	0
≥ 45	3	− 0.55 (− 0.85, − 0.24)	< 0.001	0.43	0
BMI (kg/m^2^)					
< 25	3	− 0.53 (− 0.77, − 0.28)	< 0.001	0.838	0
≥ 25	1	− 0.38 (− 0.79, 0.03)	0.069		
Group (SMD [Z-score])					
Total	6	− 0.38 (− 0.56, − 0.19)	< 0.001	0.392	3.9
Place					
Lumber spine	4	− 0.36 (− 0.59, − 0.14)	0.002	0.353	8.1
Femoral neck	2	− 0.40 (− 0.72, − 0.09)	0.012	0.169	47.2
Average age (years old)					
< 45	3	− 0.33 (− 0.56, − 0.09)	0.006	0.226	32.8
≥ 45	3	− 0.46 (− 0.76, − 0.16)	0.003	0.422	0
BMI (kg/m^2^)					
< 25	3	− 0.33 (− 0.56, − 0.09)	0.006	0.226	32.8
≥ 25	1	− 0.30 (− 0.70, 0.11)	0.151		

**Fig. 4:**
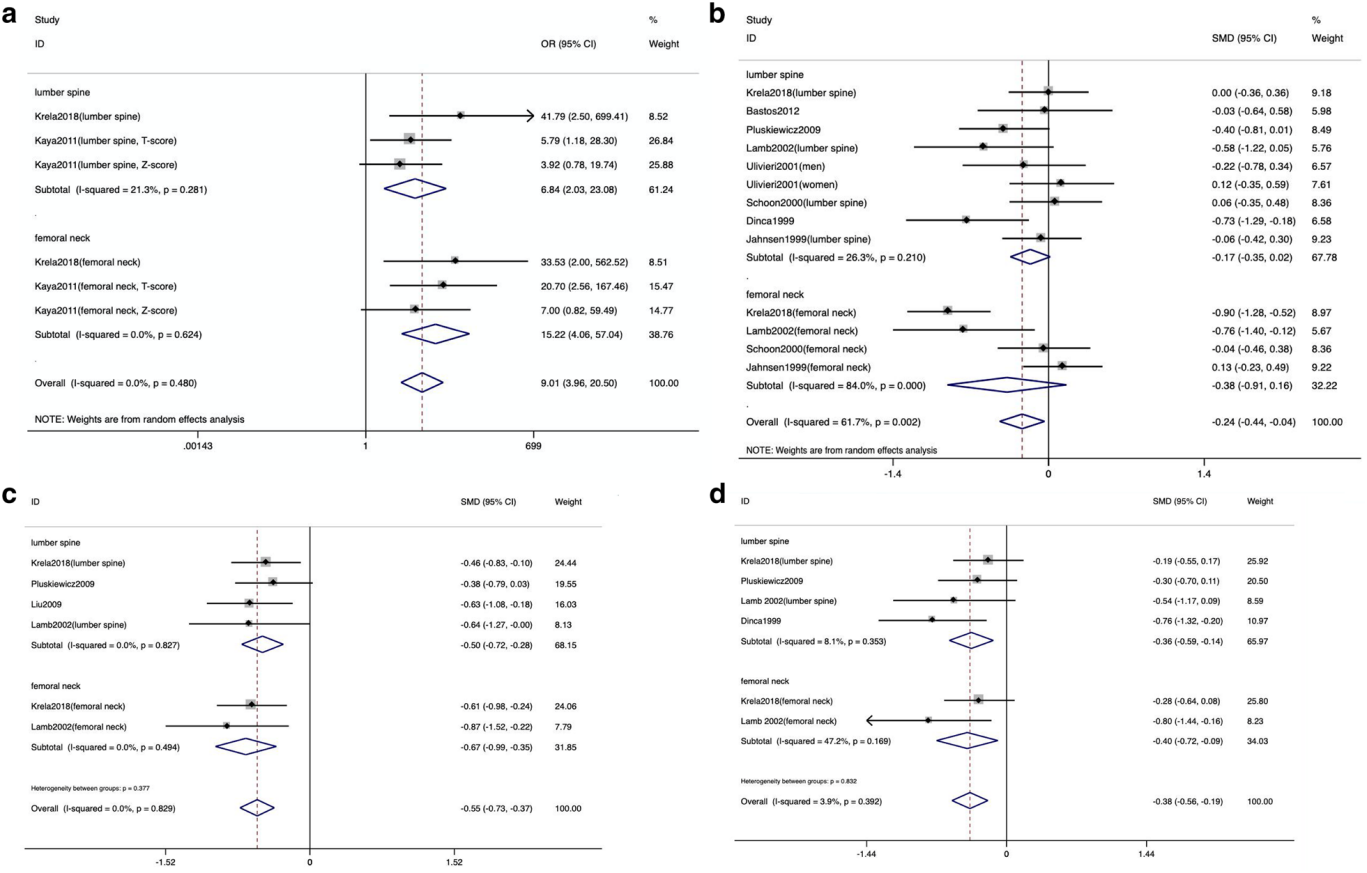
Subgroup analyses of the association between bone mineral density and ulcerative colitis in the lumbar spine and the femoral neck. **A** Pooled odds ratio (OR) for the association between low bone mineral density and ulcerative colitis in the lumbar spine and the femoral neck. Three studies did not include the outcomes based on the detection sites separately, so they were excluded from this subgroup analysis. There was no significant heterogeneity in these two subgroups (I^2^ = 21.3%, P = 0.281 and I^2^ = 0.0%, P = 0.624). **B** Pooled standardized mean differences (SMDs) for bone mineral density and ulcerative colitis in the lumbar spine and femoral neck. **C** Pooled SMDs for the association between t-score and ulcerative colitis in the lumbar spine and femoral neck. One study did not include the outcomes based on the detection sites separately, so it was excluded from this subgroup analysis. **D** Pooled SMDs for the association between the z-score and ulcerative colitis in the lumbar spine and femoral neck

The subgroup analyses also revealed a negative relationship between BMD and ulcerative colitis. Moreover, the femoral neck was more susceptible to low BMD than the lumbar spine. The results of subgroup analyses based on detection sites are shown in Fig. 4. The SMDs (95% CIs) for the bone mineral density of European people and American people were − 0.25 (− 0.47, − 0.04) and -0.03 (-0.64, 0.58), respectively. Among the group(BMD), the SMDs (95% CIs) for average age < 45 years old and ≥ 45 years old were − 0.16 (− 0.39, 0.07) and − 0.52 (− 0.83, − 0.22), respectively. And the SMDs (95% CIs) for BMI < 25 kg/m^2^ and ≥ 25 kg/m^2^ were − 0.24 (− 0.56, 0.08) and − 0.08 (− 0.31, 0.14). It is revealed that the incidence of osteoporosis in European ulcerative colitis patients was higher than that of patients in other regions, and thin or older patients were more susceptible to osteoporosis. The results are shown in Tables 2 and 3.

### (5) Assessment of bias

Publication bias was assessed using Begg’s method. All of the results suggested that there was no evidence of significant publication bias (P = 0.466, 0.200, 0.548 and 0.060).

## Discussion

Newer epidemiological studies have suggested that the incidence of ulcerative colitis might be increasing rapidly in places other than Europe [1, 2], and low BMD in ulcerative colitis patients has gained increasing attention. It has been stated that BMD is reduced in patients with Crohn’s disease but not in patients with ulcerative colitis [8]. The possible reason might be as follows: Crohn’s disease is a systemic disease with a long premorbid phase, while ulcerative colitis is a mucosal disease with an acute onset and is often limited to distal colonic tracts. In addition, Crohn’s disease also has important immunological differences when compared to ulcerative colitis [26, 27]. The localization of Crohn’s disease is in the small intestine, and intestinal resection may cause malnutrition and estrogen deficiency [28], which may contribute to low BMD. Due to these conflicting results, the present meta-analysis was conducted to identify the possible correlation between BMD and ulcerative colitis.

Four groups (OR, SMD [BMD], SMD [z-score] and SMD [t-score]) were assessed, and all of them revealed that BMD has a negative correlation with ulcerative colitis.

Several potential mechanisms may account for the association between BMD and ulcerative colitis. One of the possible mechanisms is vitamin D deficiency and secondary hyperparathyroidism. Vitamin D has been shown to have anti-inflammatory, anticancer and immune-regulatory effects, in addition to its traditional role in regulating calcium and phosphorus metabolism [29–33]. It has been reported that vitamin D deficiency is commonly observed in inflammatory bowel disease patients and is independently correlated with disease activity [34, 35]. Biochemical data from three studies [22, 24, 25] included in the present meta-analysis also demonstrated this trend: ulcerative colitis patients had lower concentrations of serum 25-hydroxy vitamin D and higher concentrations of serum parathyroid hormone. Bone metabolism is unbalanced in inflammatory bowel disease patients, with increased bone resorption but no evident variations in bone formation [36]. Another mechanism is the high circulating levels of cytokines [37]. The prevailing theory of the pathogenesis of bone loss in inflammatory bowel disease patients suggests that the increase in T-cell activity in the state of intestinal inflammation leads to an increase in the systemic release of numerous proinflammatory cytokines, such as interleukin-1, tumor necrosis factor, transforming growth factor-α, interleukin-6 and interleukin-4 [38–40]. These inflammatory factors stimulate osteoclast function, an effector of bone resorption, and could inhibit osteoblasts, a mediator of bone formation, with potential deleterious effects on BMD [41–44]. One study included in this meta-analysis [19] divided patients into three forms(mild, moderate and severe) based on the severity of disease and detected the bone mineral density respectively. And it also shown that the bone mineral density in severe patients was much lower than mild patients. This concept may explain the bone loss in ulcerative colitis patients. Moreover, other factors, such as malnutrition and malabsorption, which lead to secondary hypogonadism; corticosteroid treatment; decreased physical activity; and diminished sun exposure, may also contribute to low bone density in ulcerative colitis patients [19].

Due to the high heterogeneity in the two groups (OR and SMD [BMD]), subgroup analyses were also conducted. These analyses revealed that the femoral neck had lower BMD than the lumbar spine in ulcerative colitis patients. Additionally, thinner or older ulcerative colitis patients were more susceptible to osteoporosis, which may lead to more positive prevention in these patients. When the OR group was further divided into two subgroups based on detection sites, both subgroups exhibited low heterogeneity. This finding may explain the possible bias in the OR group. However, none of the subgroups of the SMD (BMD) group exhibited lower heterogeneity. Hence, it is possible that certain kinds of biases may not have been found. Some studies have shown that inflammatory bowel disease patients have a genetic predisposition to osteoporosis [45], such as variations in the IL-6 and IL-1 genes, which may explain the unknown bias in the detection of BMD in ulcerative colitis patients [46, 47]. Second, steroids have been shown to contribute to low BMD in ulcerative colitis patients, and various patients in these studies had taken glucocorticoids as a normal treatment for ulcerative colitis before the detection of BMD, and the doses received by these patients varied.

It is accepted that glucocorticoids can reduce BMD. Glucocorticoids not only inhibit osteoblast proliferation and the synthesis of type-I collagen and osteocalcin but also promote osteoblast apoptosis, osteoclast formation and activity, and bone resorption [49]. Moreover, glucocorticoids can also reduce intestinal calcium absorption, increase the renal excretion of calcium, and lead to an early increase in fracture risk prior to the loss of BMD [50–54]. And it is revealed that the bone mineral density of patients can significantly improve after discontinuation of glucocorticoids [55]. Glucocorticoids are conventional treatments for inflammatory bowel disease, and some patients in the studies included in the present meta-analysis had taken steroids, which may have contributed to some of the bias in the present analysis. To address this significant confounding factor, another subgroup analysis of studies on patients who had never been introduced to steroid therapy before was conducted. This subgroup analysis also exhibited a negative relationship between BMD and ulcerative colitis. The findings revealed that steroids are a factor potentially contributing to osteoporosis in ulcerative colitis patients but that ulcerative colitis itself could also contribute to low BMD regardless of corticosteroid therapy.

Another meta-analysis [48] evaluated the relationship between fracture risk and inflammatory bowel disease. However, it was reported that most fractures occurred in individuals with a BMD T-score that does not meet the conventional definition for osteoporosis (− 2.5 or lower). Hence, there might be some differences between low BMD and fractures [5]. As mentioned above, there are certain differences between ulcerative colitis and Crohn’s disease, so it would be better to analyze these two diseases separately.

The present meta-analysis has several strengths. This meta-analysis was the first to assess the correlation between BMD and ulcerative colitis. All studies were divided into four groups. These groups were separately analyzed, and certain subgroup analyses were conducted. Two groups (the SMD [z-score] group and the SMD [t-score] group) had low heterogeneity, while the OR group had low heterogeneity after the subgroup analyses. Glucocorticoids are conventional treatments for inflammatory bowel disease. The subgroup analysis of studies of steroid-free patients addressed the use of steroids, which is a confounding factor of low BMD in ulcerative colitis patients. This subgroup analysis also revealed a significant negative relationship between BMD and ulcerative colitis. Last, the large number of participants provided high statistical power. In the sensitivity analysis, the overall estimates remained significant, which contributed to these robust results.

However, there were some limitations in the present meta-analysis. First, there was significant heterogeneity among studies in the SMD (BMD) group when the data was pooled together, and this could not be explained through the subgroup analyses. Multiple factors may have caused the heterogeneity but the majority of these factors could not be examined. For example, except for the potential factors included in the subgroup analyses, genetic predisposition may also contribute to heterogeneity. But the races of people included in this analysis varied and they were not mentioned in some studies included in this meta-analysis. At the same time, it could not be excluded that some medicines such as bisphosphonates might be introduced to some ulcerative colitis patients who had severe osteoporosis. But some studies did not clarify whether the patients included had taken bisphosphonates or not. It may have also contributed to heterogeneity. Second, since the included studies were all observational studies, the severity of the disease could not be balanced, and few studies divided patients based on disease severity. This situation may have contributed to some bias in the present analysis. More convincing experimental trials should be conducted to further investigate these relationships.

## Conclusions

The present meta-analysis indicated that BMD negatively correlates with ulcerative colitis regardless of steroid therapy and that thinner or older ulcerative colitis patients are more susceptible to osteoporosis. This finding provides convincing positive implications for osteoporosis prevention in ulcerative colitis patients regardless of whether they are taking corticosteroids. More convincing studies should account for the confounding factors mentioned above to further evaluate the relationship between BMD and ulcerative colitis.

## Data Availability

PubMed, EMBASE and the Cochrane Library databases.

## Appendix

### MOOSE Checklist

Bone mineral density is negatively correlated with ulcerative colitis: a systematic review and meta-analysis.

**Table:**

**Criteria**		**Brief description of how the criteria were handled in the meta-analysis**
**Reporting of background should include**		
√	Problem definition	Newer epidemiological studies suggest that the incidence of ulcerative colitis might be increasing rapidly. Furthermore, osteoporosis in ulcerative colitis patients has gained great attention, but the epidemiologic evidence in ulcerative colitis decreasing bone mineral density remains controversial
√	Hypothesis statement	Bone mineral density is negatively correlated with ulcerative colitis regardless of steroid therapy
√	Description of study outcomes	Low bone mineral density
√	Type of exposure or intervention used	*Ulcerative colitis*
√	Type of study designs used	We included cross-sectional studies. We excluded studies of reverse association
√	Study population	We placed no restriction
**Reporting of search strategy should include**		
√	Qualifications of searchers	The credentials of the two investigators Tianyu Zhou and Jiaqi Pan are indicated in the author list
√	Search strategy, including time period included in the synthesis and keywords	PubMed from 1965—August 2019 EMBASE from 1974—August 2019 Cochrane library from 1999—August 2019 See Fig. 1 in the article
√	Databases and registries searched	PubMed, EMBASE and Cochrane library
√	Search software used, name and version, including special features	We did not employ a search software. EndNote was used to merge retrieved citations and eliminate duplications
√	Use of hand searching	We hand-searched relevant studies of retrieved papers for additional references
√	List of citations located and those excluded, including justifications	Details of the literature search process are outlined in the flow chart. The citation list is available upon request
√	Method of addressing articles published in languages other than English	We include full papers published in English
√	Method of handling abstracts and unpublished studies	We extracted information from abstracts and some abstracts which were lack of enough information were excluded. There was no unpublished study in the present analysis
√	Description of any contact with authors	None
**Reporting of methods should include**		
√	Description of relevance or appropriateness of studies assembled for assessing the hypothesis to be tested	Detailed inclusion and exclusion criteria were described in “Materials and methods” section
√	Rationale for the selection and coding of data	Data extracted from each of the studies were relevant to name of the first author, year of publication, country where the study was conducted, study population, method used to detect bone density as well as ulcerative colitis, and the number of events (or cases) and non-events (or controls)
√	Assessment of confounding	Restricted the analysis to method estimates
√	Assessment of study quality, including blinding of quality assessors; stratification or regression on possible predictors of study results	Sensitivity analyses by several quality indicators such as methods to detect *bone density* and diagnose ulcerative colitis, control selection, potential duplicate data, and origin of samples
√	Assessment of heterogeneity	Heterogeneity of the studies were explored by I^2^ statistic that provides the relative amount of variance of the summary effect due to the between-study heterogeneity
√	Description of statistical methods in sufficient detail to be replicated	Description of methods of meta-analyses, sensitivity analyses, meta-regression and assessment of publication bias are detailed in the methods
√	Provision of appropriate tables and graphics	We included 4 figures and 3 tables
**Reporting of results should include**		
√	Graph summarizing individual study estimates and overall estimate	The overall result was showed in Fig. 2 Analyses of studies on steroid-free patients was shown on Fig. 3
√	Table giving descriptive information for each study included	Table 1
√	Results of sensitivity testing	The results of subgroup analyses were showed on Tables 2 and 3
√	Indication of statistical uncertainty of findings	95% confidence intervals were presented with all summary estimates, I^2^ values and results of sensitivity analyses
**Reporting of discussion should include**		
√	Quantitative assessment of bias	Sensitivity analyses indicate heterogeneity in strengths of the association due to most common biases in observational studies
√	Justification for exclusion	We excluded studies that had not adjusted for standards, and used different assessment for the comparison groups
√	Assessment of quality of included studies	We discussed the results of the sensitivity analyses, and potential reasons for the observed heterogeneity
**Reporting of conclusions should include**		
√	Consideration of alternative explanations for observed results	We discussed that potential unmeasured confounders such as the severity of the disease may have caused residual confounding We noted that the variations in the strengths of association may be due to true population differences, or differences in quality of studies
√	Generalization of the conclusions	Bone mineral density negatively correlates with ulcerative colitis
√	Guidelines for future research	We recommend more convincing studies that could exclude the confounding factors
√	Disclosure of funding source	None

## Abbreviations

BMD: Bone mineral density
DXA: Dual energy X-ray absorptiometry
MOOSE: Meta-analysis of observational studies in epidemiology
OR: Odds ratio
CI: Confidence interval
SMD: Standardized mean difference
NOS: Newcastle–Ottawa scale
AHRQ: Agency for Healthcare Research and Quality
